# Measles: An Overview of a Re-Emerging Disease in Children and Immunocompromised Patients

**DOI:** 10.3390/microorganisms8020276

**Published:** 2020-02-18

**Authors:** Andrea Misin, Roberta Maria Antonello, Stefano Di Bella, Giuseppina Campisciano, Nunzia Zanotta, Daniele Roberto Giacobbe, Manola Comar, Roberto Luzzati

**Affiliations:** 1 Department of Infectious Diseases, Azienda Sanitaria Universitaria Giuliano Isontina (ASU GI), Via G.L. Gatteri 25/1, 34125 Trieste, Italy; stefano932@gmail.com (S.D.B.); roberto.luzzati@asuits.sanita.fvg.it (R.L.); 2 Faculty of Medicine and Surgery, University of Trieste, Strada di Fiume 447, 34149 Trieste, Italy; rma.roby@gmail.com; 3 SSD of Advanced Microbiology Diagnosis and Translational Research, Institute for Maternal and Child Health—IRCCS “Burlo Garofolo”, Via dell’Istria 65/1, 34137 Trieste, Italy; giusi.campisciano@burlo.trieste.it (G.C.); nunzia.zanotta@burlo.trieste.it (N.Z.); manola.comar@burlo.trieste.it (M.C.); 4 Infectious Diseases Unit, Ospedale Policlinico San Martino—IRCCS, L.go R. Benzi 10, 16132 Genoa, Italy; daniele.roberto.giacobbe@gmail.com; 5 Department of Health Sciences (DISSAL), University of Genoa, Via A. Pastore 1, 16132 Genoa, Italy; 6 Department of Medical Sciences, University of Trieste, Piazzale Europa 1, 34127 Trieste, Italy

**Keywords:** measles, immunity, viral–host interactions

## Abstract

Despite the availability of a safe and effective vaccine, in 2018, around 350,000 measles cases were reported worldwide, which resulted in an estimate of 142,300 deaths from measles. Additionally, in 2017, global measles cases spiked, causing the death of 110,000 people, mostly children under the age of 5 years and immunocompromised adults. The increase in measles incidence is caused by the ongoing reduction of vaccination coverage. This event has triggered public and scientific interest. For this reason, we reviewed the pathophysiology of measles infection, focusing on mechanisms by which the virus spreads systemically through the host organism. By reaching the lymphocytes from the airways through a “trojan horse” strategy, measles induces an immunosuppression status. H and F glycoproteins, both expressed in the envelope, ensure attachment of the virus to host cells and spreading from one cell to another by binding to several receptors, as described in detail. The severity of the disease depends both on the age and underlying conditions of patients as well as the social and health context in which epidemics spread, and is often burdened by sequelae and complications that may occur several years after infection. Particular attention was paid to special groups that are more susceptible to severe or atypical measles. An overview of microbiology, symptoms, diagnosis, prevention, and treatment completes and enriches the review.

## 1. Introduction

Measles virus (MV) belongs to the genus *Morbillivirus* of the family Paramyxoviridae. It is an enveloped, nonsegmented, single-stranded, negative-sense RNA virus, and its genome encodes at least six structural proteins [1].

MV (also known as rubeola virus) causes measles, an acute highly contagious infection usually seen in children. Recovery from measles is the rule but severe complications may develop in some cases [2].

Severe forms with non-pathognomonic clinical features may occur, especially in individuals with compromised or deficient cellular immunity, such as those being treated for malignant disease, transplanted, individuals with acquired immunodeficiency syndrome (AIDS), or any form of congenital immunodeficiency [3].

## 2. The Pathogen

MV virions consist of a ribonucleoprotein that is a coiled helix of protein and RNA, and an envelope that bears two types of short surface projections: Hemagglutinin (H) and the fusion (F) proteins. It is possible to differentiate between wildtype measles virus and vaccine-type virus. The gene sequences for nucleoprotein (N) and H are among the most variable of measles and are among the most used to differentiate the various measles genotypes [4]. MV owns six structural proteins (F, H, L-large, M-matrix, N, P). Among these, the N, the phosphopolymerase protein (P), and the large protein (L) are complexed with RNA. C and V, which are not structural proteins, interact with cellular proteins and are implicated in the regulation of viral transcription and replication [1]. The H glycoprotein is involved in the attachment of the virus to the host cells while the F glycoprotein is involved in the spread of the virus from one cell to another. Unlike many other paramyxoviruses, neuraminidase is not found on the envelope of measles virus [5].

The major receptor for MV is the signaling lymphocyte activation molecule (SLAM; CDw150): Wildtype virus enters mainly using this receptor, and this element explains the lymphotropism and immunosuppressive effects [6]. With regards to the receptors through which the virus can pass through the airways and then be able to spread in aerosols, the key role would be ascribed to nectin-4, an epithelial cell protein [7].

## 3. Epidemiology

Measles is seen in every country in the world and humans are the only natural host for wildtype measles virus, but monkeys may also be infected [8]. Without a vaccine, the epidemics of measles lasting 3 to 4 months could be predicted to occur every 2 to 5 years [9]. Annual measles outbreaks typically occur in late winter and early spring in temperate climates, influenced by both meteorological and social variables and by population density understood both in relation to the inhabited area and public places, such as schools [1,9]. Countries in which the measles vaccine is widely used have experienced a marked decrease in the incidence of disease [10].

Moss et al. [1] described how the high levels of viremia associated with the phase characterized by greater intense coughing coryza and therefore droplets favor the transmission of viruses. Measles has one of the highest basic reproductive numbers for a directly transmitted pathogen (defined as the number of secondary cases resulting from the introduction of an infectious individual into a completely susceptible population), significantly higher than other respiratory viruses [1,11].

With current vaccination policies, in populations that have received two doses of measles vaccine, the age distribution of measles is shifted into adolescence and adulthood [12]. In addition to this change in the epidemic curve by age, the number of measles outbreaks has increased in areas with reduced vaccination coverage and in health care settings where there is the presence of vulnerable populations that are not protected by vaccination, such as the very young, the immunocompromised, and patients with underlying immunocompromising illnesses. At this regard, in Italy, in early January 2017, 2851 cases of measles were reported: 73% with age >15 years and 27% pediatric patients, including a fatal case of a 9-year-old patient. Among the patients, 89% were unvaccinated or too young to be vaccinated [13].

## 4. Pathophysiology

**Figure 1:**
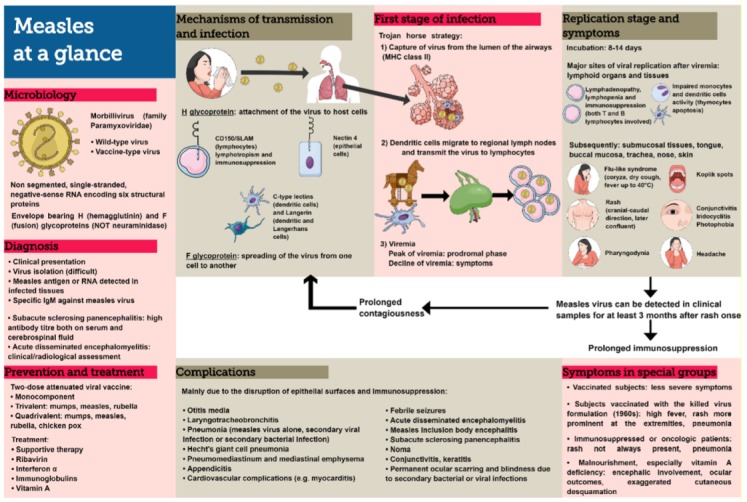
”Measles at a glance”: microbiology, mechanisms of transmission and infection, symptoms, complications, diagnosis, prevention and treatment.

MV infection starts from the luminal side of the upper respiratory epithelium, in fact the respiratory epithelium contains many other cell types besides epithelial cells, for example, immune system cells, and from here reaches the regional lymph nodes. Then, after infecting other lymphocytes, virions can also spread systemically (Figure 1).

MV binds to several receptors of the host cells. In the respiratory tract, MV attaches to the epithelial cells through the interaction of the hemagglutinin glycoprotein (MV-H) with cellular receptors. From in vitro studies, it has been seen as the MV non-wild virus (vaccine and lab-adapted virus strains) can also use CD46 (a protein that is ubiquitously expressed on all human nucleated cells) and CD150 (signaling lymphocyte activation molecule) to infect cells, but this evidence has never been demonstrated for wild strains [14,15]. Dendritic cell (DC)-SIGN and Langerin, which are two C-type lectins, function as attachment receptors for MV [16]. Furthermore, in the respiratory tract, a junction protein, nectin-4, has been identified as an additional receptor used by MV to pass through respiratory cells without causing a direct cytopathic effect [17].

### 4.1. First Stage of Infection

The initial cellular infection in the respiratory tract involves the dendritic cells and is most likely a CD150-dependent event that does not involve the epithelial cells as the primary targets for the initiation of infection. Therefore, exposure to the nebulized virus on the tonsils or adenoids is not sufficient, these stations are infected only after the onset of MV viremia [18,19].

A mechanism of cellular entry similar to that of HIV has been proposed for MV, via the “trojan horse strategy”, in which MHC class II + CD11c + dendritic cells (which are widely distributed throughout the respiratory epithelium) are used: Where the density of Langerhans cells is characterized by a progressively lower gradient from the upper to the lower respiratory tract. After viral capture from the lumen of the airway, the dendritic cells migrate to regional lymph nodes and transmit MV to lymphocytes, initiating the infection [20].

### 4.2. Replication Stage

The measles incubation phase is quite long, about 10 days before fever and 14 days before rash. The peak of viremia coincides with the prodromal phase while the symptoms are associated with the gradual viremia decline. Lymphoid organs and tissues are the major sites of viral replication and multinucleated giant cells are frequently observed in lymphoid tissues before the onset of a rash [21].

After these first sites of replication, MV also spreads to other tissues, such as submucosal tissues, tongue, buccal mucosa, trachea, nose, and skin. Viral persistence in the body has been documented mainly in epithelial cells rather than in lymphocytes that undergo a more frequent turnover [22]. During the infection, the turnover of the lymphocytes is amplified, but it is not known if it is dependent on the greater cytotoxicity of measles against the lymphocytes or their greater susceptibility to the attack of the immune system [23]. Measles RNA can be detected in clinical samples (blood, urine, and nasopharyngeal specimen) for at least 3 months after the onset of rash. This fact suggests slow clearance during convalescence [24].

## 5. Mode of Transmission

The contagiousness period is difficult to define, since the measles RNA can be detected for long periods, even months, in blood, urine, and nasopharyngeal mucosa after the onset of the rash, but the infectious period is maximum in the phase in which the virus replicates mainly in the upper respiratory tract (nasopharyngeal), rather than in the lower respiratory tract (tracheobronchial). [24,25,26]. MV is transmitted by respiratory droplets over short distances but also by small particle aerosols that remain suspended for several hours in the air [26]. The incubation period of measles is about 10–14 days, four days before rash onset until four days after its appearance [25]. The high levels of viremia associated with the phase characterized by greater intense coughing coryza and therefore droplets favor the transmission of viruses [1]. It has to be kept in mind that the contagiousness of measles is among the highest among viruses and MV can be maintained in human populations only through uninterrupted transmission chains since it does not cause known latent or persistent infectious states, nor is it detectable in animal reservoirs [27]. Measles is characterized both by seasonal epidemics (mainly in winter due to the presence of social risk factors, such as crowded enclosed places, schools opening, etc.), and by longer cycles, even more years, due to the increase in sensitive non-immunized guests during previous outbreaks [9]. There are studies showing that another important factor impacting on the cyclicality and duration of the epidemics is the birth rate [28]. In recent years, cases of measles transmissibility have also been highlighted among vaccinated patients (even with two doses) following the sharp drop in vaccination coverage, although it must be said mainly in cases of immune system abnormalities [15,29].

## 6. Signs and Symptoms

The incubation period lasts about 10–14 days, then the fever appears up to over 40 °C accompanied with cough, coryza, and conjunctivitis. Pathognomonic for this pathology are Koplik spots, which occur the day before the onset of the rash and persist for 2 or 3 days. Koplik spots appear as bluish-white lesions, slightly raised by 2–3 mm, on a reddened base and are identifiable on the oral mucosa at the level of the first molar but may also occur at the level of the soft palate and vaginal mucosa [30,31]. The early pre-rash symptoms are similar to those of other common respiratory illnesses. Gradually, the picture intensifies in 24–48 h, after which, in some cases, skin manifestations can also affect the palms. The rash lasts 3–7 days, in some cases followed by a fine scaling. An exaggerated desquamation is commonly observed in malnourished children [32,33,34]. Initially, the areas involved are those of the face, with a subsequent distribution in 2–3 days of the caudal skull (erythematous macules of 3–8 mm subsequently confluent); instead, a cough can persist for up to 10 days. Accompanying these signs are other symptoms, such as photophobia from iridocyclitis, pharyngodynia, headache, abdominal pain, and mild generalized lymphadenopathy. Based on the signs described above, a clinical diagnosis can be made: The combination of generalized maculopapular rash, fever (≥38 °C), and either cough, coryza, or conjunctivitis has a high sensitivity (75–90%) but a low positive predictive value when the incidence of measles is low, hence the need for serological confirmation [35]. Transaminases elevation is a common finding, with two thirds of measles patients experiencing it [36]. In the past, measles-associated hepatitis was thought to be related to the severity of the infection, but this has not been confirmed. Measles hepatitis is more common in children than in adults, the damage is generally hepatocellular rather than cholestatic, and is caused by a direct viral action [37,38].

### 6.1. Complications

Measles can lead to complications involving almost all organs and systems. The incidence of complication varies according to age and underlying conditions. The majority of complications is caused by disruption of epithelial surfaces and/or immunosuppression.

Measles destroys the epithelium, favoring bacterial superinfections. Cases of otitis are presented primarily due to anatomical causes of the ear structure and therefore of easy superinfection.

The same is for laryngotracheobronchitis, with a purulent exudate and evidence of secondary bacterial tracheitis, pneumonia, or both. Pneumonia is the most common severe complication of measles and accounts for most measles-associated deaths [39]. It can be caused by measles alone or can be secondary to viral infection with adenovirus or HSV (some children have the clinical pattern of bronchiolitis mediated by viruses), or secondary to a bacterial superinfection [39,40]. Measles is one of the causes of Hecht’s giant cell pneumonia, which usually occurs in immunocompromised people but can also occur in otherwise normal adults and children. About 30% of pneumonia cases are caused by bacteria, with the most often isolated pathogens being *S. pneumoniae*, *S. aureus*, and *H. influenzae*. *Neisseria meningitidis* has also been identified in some cases [41,42]. Rarely, severe pneumomediastinum and mediastinal emphysema may develop [43].

Measles-associated episodes of myocarditis have rarely been described. In the past, it was hypothesized that myocarditis cases were associated with streptococcal superinfection, but this has not been confirmed [2,44].

MV probably infects the intestinal tracts of most people affected with measles. Cases of appendicitis have been reported before and during the rash. In these cases, giant cells typical of measles were found in the intestinal epithelium [45]. In the United States, 8% of all cases are complicated by diarrhea. Particularly, in the two age groups under 5 years and over 30 years, the etiology of these cases is similar to that of diarrhea in children not infected with measles [2]. Cases of Noma associated with measles have also been described in developing countries [46].

Febrile seizures occur in 0.1–2.3% of children with measles in the United States and England [45], often without long-term sequelae. The pathophysiology is almost always that of fever and metabolic changes rather than physical alterations in the brain [47].

There are three rare but serious complications of measles that can involve the central nervous system (CNS). Acute disseminated encephalomyelitis (ADEM) is a demyelinating autoimmune disease often triggered by viral infections and occurring within days to weeks in approximately 1 in 1000 measles cases. Measles inclusion body encephalitis (MIBE) is more common in hosts with immune system disorders [48]. Subacute sclerosing panencephalitis (SSPE) is a chronic, degenerative, fatal neurologic disease that occurs on average 7 years after measles, particularly in children infected before 2 years of age, and it is almost invariably caused by wildtype virus. Wendorf et al. described an SSPE case rate of 1:1367 for children under the age of 5, but it should be noted that the highest incidence of SSPE is equal to a case rate of 1:609 for children with measles under the year of life [49]. Based on the number of cases of measles in children during 1989 to 1991, and the number of cases of SSPE reported to the CDC after those years, it was estimated that the risk of SSPE after measles is 1 per 11,000 cases, 10 times greater than originally thought. Viral genotyping revealed that these SSPE cases were caused by the wildtype measles virus circulating during those years. It appears therefore that vaccination can prevent significantly more cases of SSPE than was originally estimated [50].

Patients with SSPE have unusually high measles antibody titers in both serum and cerebrospinal fluid. The measles virus is thought to spread to the brain during the acute rash when other endothelial cells are also infected. A trans-synaptic transmission of the virus has also been hypothesized recently. Contrary to the measles virus infection of non-neuronal cells, however, in this district, there is no immediate cytopathic effect but of viral persistence. To this process, they can be responsible for the modifications of the viral proteins M-H-F. In fact, it has been described how in the presence of some antibodies to measles it is possible to modify the expression pattern of the virus genes. This could explain the reason for the higher incidence of SSPE in children infected under one year old when there are still circulating maternal antibodies [51]. Furthermore, host factors, such as defective cellular immunity and the consequent poor capacity of specific antibodies to limit the viral intracellular multiplication, are also postulated to play a role in the pathogenesis of SSPE [52].

With regards to the eye, the main complication is undoubtedly conjunctivitis, occasionally associated with keratitis, secondary to bacterial or viral infections (e.g., HSV or adenovirus), which rarely can lead to permanent scarring and blindness [53].

### 6.2. Clinical Manifestation in Special Groups

A particular situation to remember is associated with measles infection in patients partially vaccinated with 1960s inactivated (killed) measles vaccine (KMV) that sensitized the patient to measles virus antigens without providing protection. Subsequent measles virus infection leads to signs of hypersensitivity polyserositis and these developed into high fever, a rash that was more prominent at the extremities with petechiae and frequent pneumonia [2]. The form most often found in immunosuppressed, in contrast, is the one with pulmonary involvement, with or without rash [1,3]. It was noted that in cases of malnutrition, especially with vitamin A deficiency, encephalic infestations and ocular complications were more common. In these cases, it has been observed that a vitamin supplement reduces mortality [31].

## 7. Measles and Immunity

In order to understand measles’ clinical course, it is important to understand the mechanism by which the immune system can first control MV infection and subsequently prevent its reinfection.

**Figure 2:**
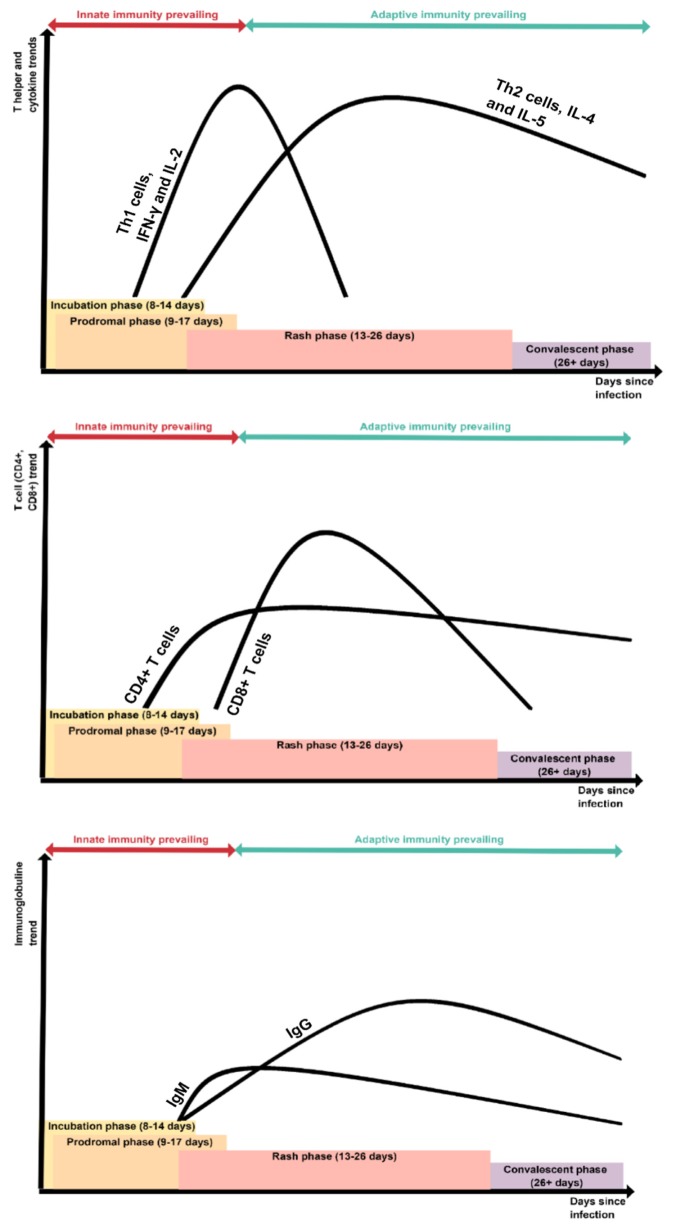
T cell, immunoglobuline and cytokine trends during the natural history of measles infection.

The innate immune component is significantly involved during the first days of infection, while the adaptive one is responsible for viral clearance and the memory development useful to prevent new infections. The antibodies production (humoral component) is certainly important in the prevention of infection with MV but the way through which it develops is not well clarified [54] (Figure 2).

In this regard, patients with mediated cellular deficit develop more severe clinical forms compared with hypogammaglobulinemic patients, supporting a leading role for the cell-mediated component [55].

Considering the results of animal studies, it is possible to hypothesize that, while the neutralizing antibody is a strong predictor of protection from diseases following exposure to MV [56], the direct role of antibodies in the clearance of the virus has not been established [57]; it must be said, however, that a role for antibody-mediated cellular cytotoxicity is possible [58].

The importance of the cell-mediated component is therefore clear, in particular the CD8+ lymphocyte response. Studies on primates have shown that even the peak of viremia and its duration were independent of B-lymphocyte deficiency if it was associated with that of the CD8+ component [59].

With regard to the role of CD4+ (whose presence has been documented from the first stages of the infection up to weeks after the disappearance of the rash), it is not well clarified. With regard to the CD8+ component, proliferation in response to MV proteins, by major histocompatibility complex (MHC) class II-restricted T-lymphocyte, has been demonstrated [59,60,61]. A Th1 cytokine profile, with elevations in peripheral blood gamma interferon (IFN-γ) and interleukin-2 (IL-2), is present during the measles prodrome [62], whereas measles convalescence is characterized by elevations in Th2 cytokines, such as interleukin-4 (IL-4) and interleukin-5 (IL-5).

Severe lymphopenia occurs during the acute phase of MV infection, involving both cytotoxic and helper T components. The mechanism by which this destruction occurs is the formation of syncytia in infected lymphocytes at the level of the lymphoid centers, including thyme, sites of the initial spread of the measles virus [63].

MV infection suppresses T-lymphocyte proliferation in response to mitogens and other alterations on the release and response to cytokines. This could explain why even the infection of a small number of these cells can have repercussions with large-scale consequences on the immune system [64].

Regarding the cytokines’ modulation, the secretion of IL-2, IL-6, IL-10, and IFN-γ is not substantially altered by the infection while the secretion of IL-4 is reduced. Reduction of IL-2Ra expression measured by CD25 expression on activated T cells has also been found [65].

Several studies focus on the role of dendritic cells, which seem to play a key role in the transient immunosuppression induced by MV infection.

Infected dendritic cells upregulate numerous cell surface molecules, including the MHC class I and II molecules. Moreover, the maturation of dendritic cells is associated with the production of chemokines that involve their migration from the circulation (where they have the role of antigenic recognition) up to the draining lymph node. This fact could allow rapid spreading of the virus to lymphoid cells [66].

As already mentioned, the syncytium mechanism is responsible for an impairment of the immune system alike. The syncytia induced by the measles virus expressing the viral glycoproteins HA and F on their surfaces are able to suppress allogeneic mixed lymphocyte reaction between dendritic cells and T cells. In fact, cell contacts between MV-infected dendritic cells and T cells and/or uninfected dendritic cells are necessary to block allogeneic T-cell proliferation [67]. The elimination of these viral surface glycoproteins on dendritic cells has been shown to enable their reactivation with a recovery of the physiological immune role.

Nonetheless, very recently, Petrova et al. [68] identified the long-term consequences of MV infection: Incomplete reconstitution of the naive B cell pool and a compromised immune memory to previously encountered pathogens. Similar results have also been shown by Mina et al [69].

After MV infection, the recovery of immunity occurs after new contact with the various epitopes, which constitutes a great risk for the patient given the naive situation. Furthermore, such a phenomenon does not allow re-creation of the same antibody variety. This phenomenon, on the other hand, is not described in animal studies after vaccination, thus, underlining how vaccination is the only useful precaution at this regard [69].

## 8. Pregnancy

MV can infect the placenta, in fact viral components have been detected in the syncytiotrophoblast [70,71]. Nevertheless, no malformative effect of the virus has been reported: The incidence of malformations observed in measles during pregnancy is comparable to that found in the general population [72]. The infection transmission to the fetus has never been documented, whereas placental damage has been described, but the precise way in which this occurs is not clear: Placental alteration explaining its dysfunction and therefore fetal death. The only cases of transmission to the fetus are associated with the phase of labor or birth [73].

No effective therapy to avoid transmission to the newborn exists but there is an indication for prophylaxis with immunoglobulins. Prevention remains the only effective weapon, and the vaccine must be administered before pregnancy [74].

## 9. Diagnosis

Classic measles with cough, coryza, conjunctivitis, Koplik spots, and a maculopapular rash that starts on the face can more easily target the measles hypothesis. Anyway, a laboratory confirmation is often useful when there are shaded shapes, special cases without exanthema, (for example, in an immunocompromised patient), or in an era (after the introduction of the vaccine) in which clinicians have less experience of this disease [1]. Measles may be diagnosed in the laboratory by viral isolation, identification of measles antigen, or RNA in infected tissues (and nucleotide sequencing can be used for precise characterization of diagnostic specimens), or demonstration of a significant serologic response to MV with the detection of specific IgM. Viral isolation is technically difficult, and core facilities for isolation are not always available. Immunofluorescent examination of cells from nasal exudates or from urinary sediment for the presence of measles antigens may be useful for rapid diagnosis of measles. More specifically, the serological method of antibody testing is the most common and simple technique used. In this context, there are various diagnostic options, some more rarely used, such as antibody neutralization or complement fixation, others more commonly used, such as the ELISA method. False-negative and false-positive results, however, may occur with ELISA, in particular the dosage of the increase in IgM title in the acute phase. However, it should be remembered that IgM can also be absent until at least 4 days after the appearance of the rash. The IgM peak is reached in a few weeks from the rash, gradually disappearing within 1–2 months after. The diagnosis can also be made by assessing an increase of at least 4 times in the IgG title between the acute phase and the convalescence phase [75].

Among the most fearsome complications, the diagnosis of SSPE can be made when a high antibody titer is found in both serum and cerebrospinal fluid [76].

Still remaining in the context of complications, the diagnosis of acute disseminated encephalomyelitis is instead commonly made by the typical clinical picture. No test is pathognomonic: Cerebrospinal fluid examination usually reveals a lymphocytic pleocytosis and raised protein levels both greater than those typically found in multiple sclerosis. The cerebrospinal fluid oligoclonal band presence is less common in acute disseminated encephalomyelitis than in multiple sclerosis. Computed tomography images may be of little significance while the brain magnetic resonance imaging showing the presence, in the T2-weighted sequences, of multiple hyperintense areas that have characteristically indistinct margins and assume the contrast medium is useful for diagnosis (magnetic resonance imaging performed too early may be not very characteristic) [77].

## 10. Vaccine

There are various types of measles vaccines, the monocomponent is used in most African countries and in others, including Russia [78]. The combined mumps, measles, rubella (MMR) vaccine is used instead in the rest of Europe and North America. A combined quadrivalent of mumps, measles, rubella, and varicella (MMRV) is also available in the United States and presents safety and immunogenicity similar to the MMR vaccine [79], although the risk of febrile convulsions is higher compared to the trivalent one.

All these vaccines are attenuated viral vaccines that replicate within the host to induce protective immunity. The World Health Organization recommends the first dose to be given at 12 months and the second dose at 15 to 18 months [80]; the CDC-recommended schedule in the United States is to administer the first dose at age 12 to 15 months and the second dose at age 4 to 6 years [81].

In some special cases, the WHO recommends the first dose of measles-containing vaccine (MCV1) to be administered at 9 months of age in contexts with endemic measles. In special situations, such as during outbreaks, the first dose can be anticipated at 6 months, the same for displaced populations or children with HIV infection [80].

However, some studies have shown that in the two-dose vaccination program, when the first dose is administered before the age of 15 months, the susceptibility to measles infection is higher at up to 2–4 fold [82]. In this regard, it should be added that there is evidence of how, if the first dose is given before or after the age of 9 months, there is a lower antibody titer but the vaccination efficacy is not inferior, as the T-cell response is independent of vaccination timing. In any case, the WHO recommends two doses of vaccine when the first is administered before 9 months of age [83].

To achieve protective efficacy (herd immunity), a level of immunity is required in the entire population. Precisely, the required vaccination coverage against measles ranges from 93% to 95% with two doses of measles vaccine [27,84].

With regard to the different types of vaccine, it has been shown that the measles-type wild virus and the attenuated vaccine virus activate different immune cascades (NFkB/NLRP3 signaling without an interferon type 1 response to the wild virus, related interferon for the vaccine virus), but the implications of these differences for the development of adaptive immunity are unclear [85]. Anyhow, the currently accepted measles protection correlates to a titer of neutralizing antibodies specific for MV >120 mIU/mL [56,86,87].

The role of B cells as a response to the vaccine is also not completely understood. Memory B cells are important for a prompt humoral response after contact with the wild virus but most likely this activation is mainly due to the plasma cells activated by the antigen in the bone marrow rather than from memory B cells [88,89].

As described by Haralambieva et al., the response to the vaccine is impacted by both host genetics and environmental factors. Regarding the genetics involved in protective immunity against measles, there are highly polymorphic genetic variants of HLA and non-HLA [90].

### Adverse Effects of Vaccination

The adverse effects of the measles vaccine are extremely infrequent and in most cases with minimal clinical impact and self-resolving.

Table 1 compares the incidence of the main complications of measles infection and that of side effects after the measles vaccine [91].

## 11. Therapy

There is no current standardized therapy for measles. However, often, some antivirals are used alone or in combination. An example of this is ribavirin and interferon α. Indeed, there is little literature on therapy and the same indications are mainly based on clinical cases; most of them support ribavirin, especially used at higher doses [92,93]. As a possible further therapeutic approach, there is that of the administration of immunoglobulins, especially those specific for measles [94]. Other clinical evidence would support the use of vitamin A, since it would play a role in promoting epithelial cell turnover, in particular in the respiratory tract and in the intestinal district. The effect of vitamin A is more evident in children under the age of 2 years, although anecdotal cases are also described for adults. The dosage is not well defined, although it would seem that repeated 200,000 IU have clinical effects. Of note, there is no consensus on the dosage of ribavirin. Reported cases were treated with a dosage of 50 mg/kg/day at 1 g every 6 h with or without loading doses. The role of steroids is also discussed, whose utility is described in some case reports of measles pneumonia [95,96,97,98].

## 12. Summary and Future Directions

Various viruses maintain their pandemic role through the duration of the clinical and pre-clinical contagious phase, being able to recur and hit the population several times, eluding the immune response by not giving a permanent antibody response through minor and major mutations (shift and drift) from year to year. Measles, while producing an immunizing immune response, instead persists globally mainly due to other mechanisms: Primarily, the very high infectivity and prolonged prodromal phase with a long period of preclinical viral shedding.

In fact, the measles virus in the early stages of infection, through interactions with antigen-presenting cell proteins, inhibits the transcription and translation of genes coding for IFNs, thus resulting in a longer lasting preclinical phase. During this period, the patient is contagious even without having yet expressed the clinical characteristics, which are manifest only after the inflammatory signaling cascades are activated and plasma levels of NFκB-induced proteins IL-6 and IL-8/CXCL8 and inflammasome products IL-1β and IL-18 are increased [90]. The rapid spread of the virus in various areas of the body is favored by the target cells that transport it to the various areas of the body and by the fact that the immune response is altered early. Mechanisms by which the virus evades the immune system are twofold. On one hand, it directly damages the T helper response whereas, on the other hand, it acts as a dysregulator of the chemotactic response.

Recently, it has also been shown how MV can favor complications and superinfection in the long term and not only contextual to the viral infection. Furthermore, it must be added that being an extremely infectious virus, respiratory isolation measures must be used in suspected or ascertained cases.

Nowadays, with the reappearance of epidemic outbreaks, it must be stressed that, in all cases of immunosuppressed and apparently immunocompetent vaccinated patients (especially with a single dose), the clinical picture can be shaded, meaning that it does not necessarily include the rash and other pathognomonic elements; therefore, a hypothesis and clinical diagnosis may be more difficult but must be taken into consideration.

In any case, as it has been said, there are no effective therapies even if the disease is found or suspected in the early stages. So, it remains clear that the only system currently available and extremely effective for reducing or eliminating the circulation of the measles virus is the vaccination. It should be remembered that the vaccine administered in two doses confers immunity to measles to approximately 97% of the population. The remaining 3% can still contract one of those diseases, although often with milder symptoms [81].

While decades ago, the anti-vaccine movements were mainly supported by religious issues, in the late 1990s, following a publication on the Lancet Journal (later withdrawn) in which correlations between vaccines and autism were highlighted, and anti-vaccine movements for alleged safety issues began to increase. This phenomenon has therefore been supported by ideas about neoliberalism and parental decision-making overriding medical experience.

A further problem is that several famous people or politicians declare themselves not in favor of vaccines, allowing the spread of the anti-vaccine movement.

In addition, the presence of numerous websites and forums on social networks contribute to the increase of the anti-vaccine support for the sole advertising and economic interests. Thus, it means that it is mandatory to intervene at different levels of society, not only with vaccination policies but also with actions aimed at these public and unsupervised media platforms [99].

First, policies of greater control regarding fake news and the deletion of forums on social media that could put public health at risk have been proposed and already partially implemented.

In this regard, it has been highlighted how a careful and precise statistical description of the side effects of vaccines (most of the times mild and non-dangerous reactions) with respect to the possible preventable complications of the diseases can be useful.

Furthermore, public health organizations should publish articles online or in successful booklets about vaccines and horror stories from preventable diseases. Some parents and famous people, who strongly believe in the importance of vaccines, can act as “vaccine ambassadors” to encourage peer-to-peer communication as interventions [100].

A further hypothesis to be considered and implemented, at least in the areas already with the lowest vaccination coverage, is that of compulsory vaccines for enrollment in school. To reach this goal, research by many professional figures like sociologists, psychologists, public health researchers, needs to be integrated with strategies launched by nonprofits, state-level health initiative, and community health promotion efforts.

## Figures and Tables

**Table 1:** Comparison between main side effects of measles vaccination and main complications of measles infection. Adapted from Bester [91].

Measles Vaccination		Measles Infection	
Total side effects per 1 million doses administered	0.0033%	Total complications per 1 million of infected children	30%
Main side effects		Main complications	
Joint pain	25%	Diarrhea	8%
Fever	5–15%	Otitis media	7%
Rash	5%	Pneumonia	5%
Febrile seizures	0.3–0.8%	Primary measles encephalitis	0.1–0.3%
Thrombocytopenia	0.003%	Acute postinfectious encephalomyelitis	0.1%
Anaphylaxis	0.0001%	Subacute sclerosing panencephalitis	0.01%
Encephalitis	0.00002%	Death	0.2%
Parotitis	---		
Lymphadenopathy	---		
